# Comparing methods for scaling shape similarity

**DOI:** 10.3934/Neuroscience.2019.2.54

**Published:** 2019-05-05

**Authors:** Ernest Greene

**Affiliations:** Laboratory for Neurometric Research, Department of Psychology, University of Southern California, Los Angeles, California, USA

It is useful to evaluate alternative methods for quantifying shape attributes to see which best predicts human judgments of shape similarity. Any insights that might be garnered would contribute to our understanding of visual mechanisms, and would also have potential applications in artificial intelligence—more specifically in machine vision. This brief commentary provides a recap of recent work from my laboratory that was directed to the attention of the artificial intelligence community [1] but which should also be of interest to those who study biological vision. It compared two computational methods for scaling similarity of two-dimensional shapes, followed by an experiment that determined the degree to which the scale values predicted human judgments of similarity. The results are at odds with a classic concept that proximity of neighboring contours is a prime factor in the perception of shape similarity.

Procrustes analysis was one of the methods used to assess shape similarity. Procrustes analysis is a statistical method for comparing shape pairs that normalizes distances of boundary markers relative to their centroids, centers the shapes on their centroids, and then derives the minimum Euclidean distance among all pairs of markers. It has strong mathematical roots [2]–[5], and has been applied in various engineering and scientific fields, including classification of rock formations [6], and classification of facial features [7]. In biology it is known as geometric morphometrics [8],[9]. A comprehensive overview of alternative implementations is provided by Dryden & Mardia [10].

**Figure 1.**
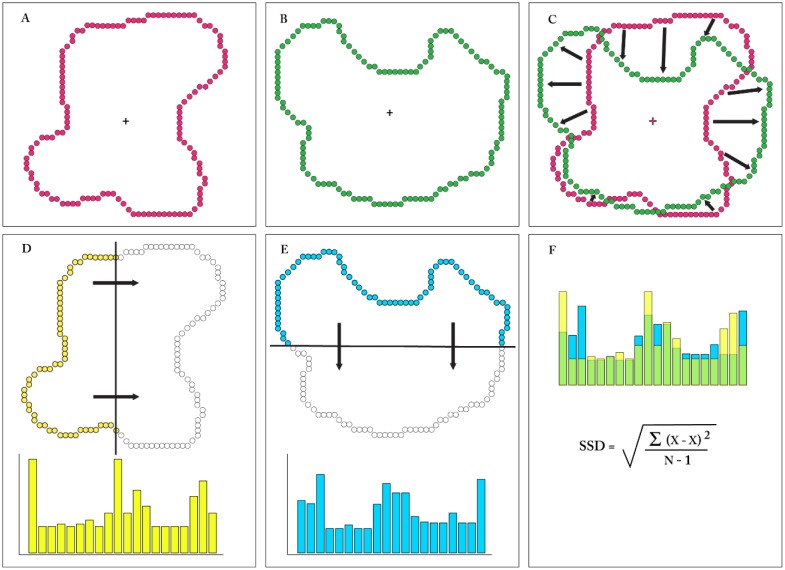
The upper panels of the figure illustrate the Procrustes method. Panels A and B show two shapes that have an equal number of boundary markers (dots). The centroid for each shape is shown with a plus sign. Panel C shows the shapes superimposed, and centered on the centroids. The arrows represent spans between the boundary markers, as required by the Precrustes method. The mean across all corresponding markers around the boundary would be calculated, and this process repeated with stepped pairing to determine the minimum mean span. This value provides an assessment of the similarity of the two shapes, the smaller the value, the greater the similarity. Panels D and E illustrate the scan method for summarizing the two shapes. Each shape is scanned with both a horizontal and vertical sweep by polling waves, though in each panel only a partial scan in one direction is illustrated. A polling wave provides a successive count of the number of boundary markers being encountered, and these values are further processed to provide a histogram that serves as the shape summary (as shown below each shape). Panel F shows comparison of the shape summaries using a sum-of-squared differences calculation, the resulting value providing a measure of shape similarity. A smaller value is provided by greater overlap of the histograms (green), with a smaller value indicating greater similarity of the shape summaries.

For comparing 2D shapes formed as outline boundaries, one must discretize the perimeter to provide an equal number of location markers, as illustrated in Figure 1. The analysis requires an iterative assessment of spans between marker pairs at all perimeter locations. One arbitrarily chooses a marker from each shape, assesses the span between them, then steps to the next adjacent pair to assess that span, then the next, and so forth until all pairs have been evaluated. The mean of these spans is calculated, and that becomes a candidate similarity value. Passing through all of the pairs is not sufficient, however, for the starting pair that were chosen might not provide the minimum mean value. So one returns to the starting point on one shape, pairs with the marker position on the other shape that is one step away from what was previously used, and repeats the loop around the boundary to derive another candidate mean value. This iterative process continues until all combinations of marker locations have been measured, after which the lowest mean value is chosen as reflecting the degree to which the two shapes are similar. A comprehensive Procrustes analysis could include adjustments for rotation and size, but here those steps were not applied because the size and orientation of target shapes remained unaltered for human judgments of similarity.

The second method for scaling similarity is based on the novel concept that shape encoding has evolutionary roots in motion processing by the retina or optic tectum. Motion of the object or eye can register the encounter of contours in the image, and waves within a neural network might be used to register stationary contours. One can model the concept with polling waves that pass across a shape, providing a successive count of the number of boundary markers that are encountered. These counts provide bin values of a histogram.

Greene & Patel [11] used polling waves that passed across each shape in the horizontal and vertical directions, and combined these in tandem into a single histogram. To adjust for differences in shape size, the raw histograms were re-binned and normalized to provide a 20-bin “summary histogram” for each shape. Similarity values were derived using a sum of squared differences calculation for each of the summary histograms. The scan registration and comparison steps are illustrated in the lower panels of Figure 1.

**Figure 2.**
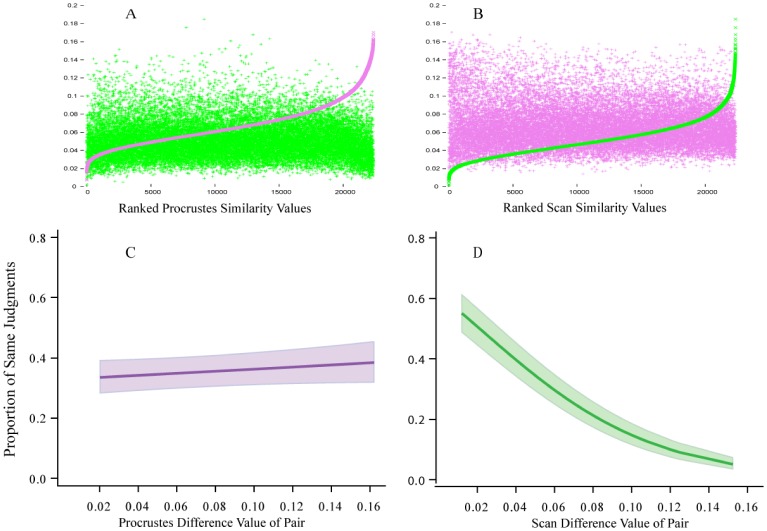
In panel A the pairs have been ranked according to the size of the Procrustes similarity values, wherein the sigmoid function (purple) provides a Procrustes scale of shape similarity. The corresponding scan similarity for each of the pairs is shown with green tokens. Panel B shows the reverse. The size of a given Procrustes value appears to be unrelated to the size of the corresponding scan value, and this was confirmed by a lack of correlation (see text). Panels C and D show binomial regression models and confidence intervals for perceptual judgments in a match-recognition task. The size of the Procrustes values did not predict the probability that the shape pairs would be seen as similar, but the scan values provided significant prediction of similarity judgments.

An inventory of 480 unknown shapes were used for scaling of similarity, as this avoids complications that might be provided from long-term memory. Each shape consisted of an outline boundary formed by a continuous string of discrete dots (see Figure 1). Procrustes similarity values were derived from the pairs that were approximately the same size (then adjusted to have equal dot counts), and these were ranked and appear as the purple-colored sigmoid in Figure 2A. That panel also plots the scan similarity value that corresponds to each of the ranked Procrustes values, which appear as the widely scattered pattern of green tokens. Panel B shows the reverse, wherein the green sigmoid provides the ranked scan-similarity values and the Procrustes values are widely scattered purple tokens. A lack of correspondence for the two similarity measures is suggested by the plots themselves, and this was confirmed by finding the correlation of the ranked values to be −0.14. This small negative correlation indicates that the two methods are assessing different shape attributes.

An assessment of the degree to which the scale values predicted human judgments of similarity was conducted using a match-recognition protocol [12]. The basic task uses an inventory of unknown shapes, each formed as a continuous string of discrete dots, like the examples shown in Figure 1. Each shape is briefly displayed as a target only once. This is quickly followed by display of a comparison shape that is a low-density version of the target or a low-density version of a different shape. The respondent judges whether the comparison display matches the target, or not, by saying “same” or “different.”

The use of low-density comparison shapes is required to prevent perfect performance, which would the outcome if the full complement of boundary markers were provided by the comparison displays. The judgment can be described as “match recognition” though it is clear that the low-density version of the target is not identical to the target. It is more accurate to say that the respondent is judging the similarity of the shapes that were provided by the target and comparison displays, and that works well for assessing which of the similarity scales provides the best prediction of human shape judgment. This can be done by assessing whether the size of the scale value predicts the probability that respondents will judge a given pair combination to be the “same” or see them as “different.” So here the comparison shape is never a low-density version of the target, per se, but the degree to which a respondent judges a pair member as being the same as the target reflects the similarity of the two shapes.

A random sample of 320 pairs was chosen from the ranked combinations, and 20 respondents judged whether a given pair displayed the same shape. The Procrustes and scan methods had provided similarity scale values for each of the pairs, so the question was how well those values predicted the probability of “same” judgments. This was assessed with binomial regressions, which are shown in the lower panels of Figure 2. It is clear from panel C that the Procrustes scale values do not predict the probability that the pairs would be seen as similar, and the correlation of the two measures was essentially zero. The binomial regression for the scan-similarity values against probability of same judgments (panel D) was significant at *p* < 0.0001, and the correlation of the two values was 0.40.

It is somewhat surprising to see that the Procrustes method does not predict human perception of similarity. The concept that similarity would be determined by the relative proximity of adjacent locations around the boundary is intuitively appealing, and the calculation of spans provides an appropriate implementation of that concept. The critical failure may be that the method is intrinsically local. Biological vision may draw more heavily on the global relationships that are present in a given shape, this being the view advocated by Gestalt theorists. The scan method converts the relative positioning of boundary markers into bin counts, wherein the markers at various locations along the boundary are included at different locations within the summary histogram. A given bin reflects not only how many boundary markers were encountered by a scan wave, but also the location of that portion of the boundary relative to the full span of the shape. Finding that the scan-similarity measures correlate with human judgments of similarity provides support for the proposition that 2D shapes are summarized using scan waves that convert the shape into a 1D message that can be more readily transmitted and stored as an information packet.

Scan encoding is based on movement of the polling wave. A number of laboratories have proposed that motion is involved in the encoding of contrast information, especially that provided by contours. Greene [13] suggested that spreading waves generated by retinal polyaxonal amacrine cells may be involved in the encoding of shape information. Gollisch & Meister [14] proposed that retinal ganglion cells are synchronously activated by local contrast at the end of saccadic eye movements. Ahissari & Arieli [15] as well as Rucci & Victor [16] argue that the drift that occurs between fixations can contribute to contour detection.

There are numerous alternative ways that scan waves might be used to encode boundary marker locations, and the findings described above do not provide firm evidence that the visual system makes use of scan encoding. However, new concepts for shape encoding are needed [17], and these results suggest that the scan concept should be further investigated.
